# Accelerating metagenomic read classification on CUDA-enabled GPUs

**DOI:** 10.1186/s12859-016-1434-6

**Published:** 2017-01-03

**Authors:** Robin Kobus, Christian Hundt, André Müller, Bertil Schmidt

**Affiliations:** Institute of Computer Science, Johannes Gutenberg University Mainz, Staudingerweg 9, Mainz, 55435 Germany

**Keywords:** Metagenomics, Taxonomic assignment, Exact *k*-mer matching, CUDA, GPUs

## Abstract

**Background:**

Metagenomic sequencing studies are becoming increasingly popular with prominent examples including the sequencing of human microbiomes and diverse environments. A fundamental computational problem in this context is read classification; i.e. the assignment of each read to a taxonomic label. Due to the large number of reads produced by modern high-throughput sequencing technologies and the rapidly increasing number of available reference genomes software tools for fast and accurate metagenomic read classification are urgently needed.

**Results:**

We present cuCLARK, a read-level classifier for CUDA-enabled GPUs, based on the fast and accurate classification of metagenomic sequences using reduced *k*-mers (CLARK) method. Using the processing power of a single Titan X GPU, cuCLARK can reach classification speeds of up to 50 million reads per minute. Corresponding speedups for species- (genus-)level classification range between 3.2 and 6.6 (3.7 and 6.4) compared to multi-threaded CLARK executed on a 16-core Xeon CPU workstation.

**Conclusion:**

cuCLARK can perform metagenomic read classification at superior speeds on CUDA-enabled GPUs. It is free software licensed under GPL and can be downloaded at https://github.com/funatiq/cuclark free of charge.

## Background

Metagenomic read classification is a common and increasingly important bioinformatics technique where short DNA reads stemming from a genomic sample are automatically annotated with a taxonomic label such as species or genus identifiers by mapping them onto a large set of reference genomes. Metagenomic sequencing studies are gaining popularity with prominent examples including the sequencing of human microbiomes [1] and diverse environments like seawater [2] in order to analyze their composition and to study their temporal variation. Precise knowledge about the individual components of a sample enables researchers to understand the interaction with surrounding environments or to gain information about the integrity of the analyzed system.

With DNA sequencing becoming faster and cheaper, the amount of recorded data is expected to rapidly grow in the foreseeable future. As an example, online services such as *μ*Biome (see https://ubiome.com) are currently performing the sequencing and analysis of a human gut sample for less than US$ 100. Thus, the design and implementation of exceedingly fast and precise metagenomic read classification algorithms remains an important topic for academic research and commercial solutions.

A number of approaches have been proposed to solve the read classification problem by employing machine learning techniques on top of nearest neighbor information between probed reads and reference genomes. BLAST [3] and BLAST-based methods like MEGAN [4] use inexact alignment of the sequences. PhymmBL [5] uses BLAST in combination with probabilistic models, while NBC [6] examines the composition of a sequence in consideration of Bayes’ rule. Unfortunately, these approaches are too slow for classifying the huge amounts of reads produced by current *next-generation sequencing* (NGS) technologies.

The recently introduced Kraken [7] and CLARK [8] methods represent a major improvement in terms of classification speed (running almost three orders-of-magnitude faster than alignment-based approaches such as MegaBLAST). Both tools analyze each read by querying its *k*-mers against a pre-built database. In case of Kraken, this database contains all *k*-mers of the considered reference genomes, which are subsequently mapped to their *lowest common ancestor* (LCA) in a taxonomic tree. For each input read a subtree containing all its *k*-mer’s LCA taxa is constructed and examined to classify a read. CLARK employs a pre-built database of target genomes exclusively consisting of unique *k*-mers. Ambiguous *k*-mers shared among multiple targets are ignored. Note that a target can consist of multiple genomes, e.g. by treating genomes from the same genus as a single target. Finally, the best fitting candidate of the target genomes is used to label the input sequence.

CLARK and Kraken can be run in several user-defined classification modes. For example the *full mode* of CLARK provides a complete analysis of the input sequences and therefore reaches the highest sensitivity. Further it provides confidence scores, which allow to abstain from uncertain classifications thus increasing precision. The remaining modes skip parts of the work to speedup the classification procedure at the cost of sensitivity. Apart from the aforementioned modes the user can specify a sampling factor controlling the ratio of target *k*-mers to be considered during classification.

A recent benchmark study [9] showed that *k*-mer based approaches performed among the best in a comparison of 14 tested tools in terms of both read assignment accuracy at genus/phylum level and classification speed. Kraken and CLARK can process NGS data with a speed of around 1 million reads per minute (depending on the read length) on a single CPU core. However, as metagenomic sequencing transcends from research labs to clinical and industrial applications even higher processing speeds are needed. For example, the estimation of species abundance in DNA sequences from a metagenomic sample usually requires a statistical method running on top of a read-level classifier where this classifier needs to be called excessively as a subroutine [10].

*Graphics processing units* (GPUs) can provide up to one order-of-magnitude higher peak performance compared to CPUs through massive fine-grained parallelism at a highly competitive price-performance ratio. Using programming languages such as CUDA, they can be used for general-purpose applications. Successful examples of GPUs applied to NGS read analysis include read mapping [11], RNA-Seq spliced alignment [12], error correction [13], *k*-mer counting [14], and assembly [15]. In this paper, we show how CUDA-enabled GPUs can be used to accelerate metagenomic read classification. The limited size of video RAM attached to a GPU makes the storage of large databases challenging: they have to be distributed among several GPUs or split into smaller parts that are queried successively. Among the two considered methods (Kraken and CLARK), CLARK uses a smaller database and further allows to partition the database into smaller parts, which can be queried in batches. As a result, we have chosen the CLARK method as candidate for our GPU parallelization.

This work presents the design and implementation of computation schemes for massively parallel accelerators in order to accelerate read classification. Our software tool, cuCLARK, features support for multiple GPUs and further provides a light version allowing for the execution on legacy workstations with only 4 GB of RAM and 1 GB of video RAM. Using a number of datasets we show that cuCLARK running on a single Titan X GPU can classify up to 50 million reads per minute. It outperforms multi-threaded CLARK for species- (genus-)level classification running on a high-end 16-core Xeon E5-2683 v4 CPU by a factor between 3.2 and 5.1 (3.7 and 5.2) including all data transfers over the relatively slow PCIe bus, which is often a bottleneck for accelerators. Note that classification results of cuCLARK and CLARK are identical except for the ordering of equally scored targets.

## Implementation

cuCLARK provides similar functionality and usability to CLARK producing compliant output to CPU-based CLARK’s *full mode*. It produces output in the same format and thus enables the user to take advantage of the post-processing scripts bundled with CLARK. Moreover, cuCLARK provides the same command line flags specifying common parameters such as *k*-mer length and database sampling factor in order to facilitate the easy integration into already existing bioinformatics pipelines.

In contrast to that, a number of implementation details of CLARK’s classification algorithm had to be modified. In order to fully utilize the compute capabilities of a modern GPU, the execution needs to be highly parallel and make use of all its available resources. Efficient memory access and management are key for accelerating read classification, since the algorithm is dominated by random accesses to a large reference genome database. This database typically exceeds the amount of memory available on a single GPU. cuCLARK supports classification on a workstation with a single GPU and compute nodes with multiple GPUs.

Despite the differences in implementation, CLARK and cuCLARK produce the same target scores for each input object. The only discrepancy in the output derives from the case where multiple targets reach the same score for the same object. It can therefore happen that the programs choose different assignments if two or more targets reach the highest score. In these cases it is not possible to determine which target is the right one to choose as classification result and cuCLARK (as well as CLARK) reports the same lowest possible confidence score (which depends on the two highest target scores). Users may request the extended output which includes all target scores for each object for further analysis.

### Database construction

Initially, a database is build in a pre-processing step. This is achieved by creating an index of all *k*-mers contained in the target (*reference genome*) sequences and collecting their number of occurrences per target. After all targets have been processed, any *k*-mers that are shared by multiple targets are removed from the index. The remaining *k*-mers are therefore called *target-specific* or *discriminative*. In the classification stage, the *k*-mers of the input objects (*reads*) are compared to these specific *k*-mers.

The database construction method of cuCLARK modifies the CLARK database structure as follows. Instead of storing each *k*-mer or its reverse complement depending on which appears first, we always choose the canonical *k*-mer which is the lexicographically smaller of the two. This choice is also done for each *k*-mer of a query read during the classification stage. Instead of querying the *k*-mer first and the reverse complement in case of a miss, we always determine the canonical *k*-mer, which helps to avoid branch divergence of the parallel execution. The database files which are stored on disk have the same format as CLARK’s and can be used instead of its own. Note that, cuCLARK’s database files can be used with CLARK but not vice versa.

**Fig. 1:**
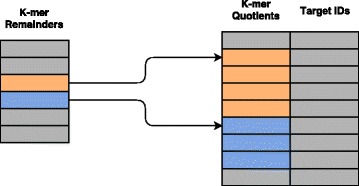
Database Structure of cuCLARK. The quotients of the discriminative k-mers and the corresponding targets are loaded into *one large array* each. For each k-mer remainder we calculate the pointer to the first element of its hash table bucket

While the creation process of the database is almost the same as in CLARK, we have changed the database pre-loading for classification. The hash table of CLARK consists of key-value pairs in a vector for each bucket. The buckets are themselves stored in a large vector. This nested container format is unsuitable for CUDA accelerators. Our approach uses an array for all keys and another one for all values. Values are accessed by pointers calculated by a prefix sum of the bucket sizes from the original hash table. This not only enables fast loading because the database files are already laid out the same as the arrays, it also allows for cache friendly traversing of consecutive keys when querying the database. This compressed form of the hash table is possible because the buckets do not change in size after their initial construction. Our data layout provides an additional advantage: If the database does not fit into the memory of a single GPU, it will need to be split up into several parts. This can be easily achieved by dividing the arrays in chunks and calculating the value pointers accordingly. Figure 1 shows the database structure of cuCLARK.

We use page-locked host memory to store the database in RAM to improve the transfer bandwidth to the graphics memory over the PCIe bus. The program is able to make use of multiple accelerator cards and adjusts the size of the database chunks according to their memory sizes. If the combined memory of all available cards is not sufficient to hold the whole database, we have to query the parts in separate stages.

### Classification process

Before we can put the GPU to work, the input files need to be processed by the CPU. CLARK divides the input by the number of CPU threads if executed in multi-core mode. We have taken the idea one step further and partition the files into a user-defined number of batches. It is advisable to choose a multiple of the number of threads in order to achieve good load balancing. The benefit of smaller batches is a lower memory consumption per input chunk. This enables us to use most of the graphics memory for the database itself when processing batches on the GPU.

FASTA or FASTQ are accepted input file formats. First the length of the contained DNA sequences is determined in order to allocate sufficient memory for both input sequences and classification results. We again use page-locked host memory to achieve higher throughput.

Next, base-pair symbols {A,C,G,T} are encoded into a two-bit representation. If a sequence contains an ambiguous character (N) it is split into the parts preceding and following this character. Sequences or sequence parts which are shorter than *k* and therefore do not contain any complete *k*-mer are disregarded. Next, the sequence data of a batch is transferred to graphics memory for classification.

**Fig. 2:**
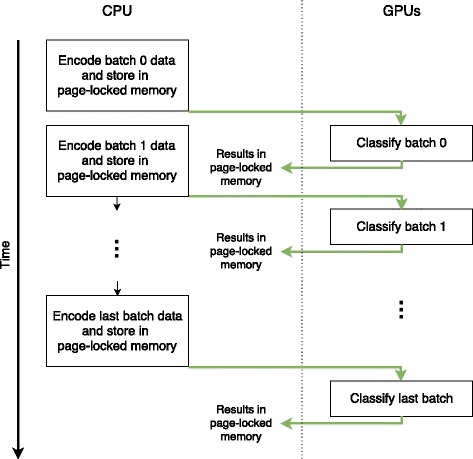
Asynchronous execution on the GPU. The processing on the GPU can be performed asynchronously to the CPU. Memory transfers (*green arrows*) and classification are handled by the GPU while the CPU already prepares the next batch

Aside from higher transfer speeds the use of page-locked memory allows for batches to be processed asynchronously by the GPU. This means after a CPU thread has prepared all sequence data of a batch, the batch is scheduled for classification on the GPU and the CPU can continue with the next batch without having to wait for the GPU to finish (see Fig. 2).

**Fig. 3:**
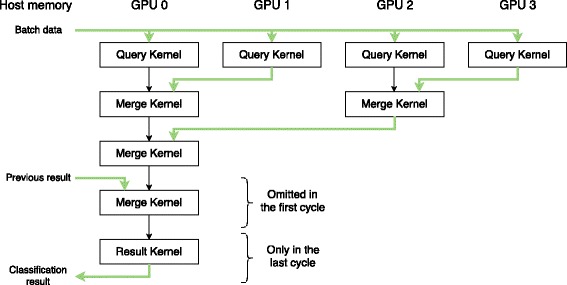
Classification cycle for a single batch. Each GPU queries the batch against a different database part. Then we merge the query results of the individual GPUs with the result of the previous cycle. After we merged all results in the last cycle, we can finally get the classification result. All kernels are explained in Section CUDA implementation

The classification process consists of multiple steps. First each GPU queries the batches against the database part it currently holds. Subsequently, the results of all individual GPUs are collected. If the database doesn’t fit into graphics memory in one piece, we swap database chunks and query the batches again. The number of cycles we need depends on the database size and the amount of available GPU memory. After the whole database has been consulted and the results have been merged, we find the two best targets for each NGS read. Identical Analogous to CLARK, we use the target with the most hits to classify a read and calculate the confidence score from the hit counts of the best and second best target. Figure 3 illustrates the classification cycle for a single batch on four GPUs.

### CUDA implementation

The CUDA programming model requires the use of kernels, which can be executed in parallel by a large number of threads on the GPU. The threads are grouped in blocks, where each block has access to a fixed amount of shared memory. This fast but small memory can be accessed simultaneously by all threads of a block for caching data from the global graphics memory or to store intermediate results. A significant speed-up can be achieved compared to repeatedly accessing the same global memory region. The drawback of the limited amount of shared memory is that less blocks can be processed concurrently if they require too much memory each. In the following we describe the CUDA kernels we have implemented for the classification described above.

#### Query kernel

The query kernel uses one thread block for each sequence (NGS read). The threads in the block first initialize the counters for all targets (reference genomes) in shared memory. Subsequently, they load the sequence data into shared memory in a coalesced manner. CUDA threads construct consecutive *k*-mers from the sequence, where each thread is responsible for a single *k*-mer at a time. Each thread compares the *k*-mer with its reverse complement to determines the canonical *k*-mer. The resulting *k*-mer is queried against the database.

**Fig. 4:**
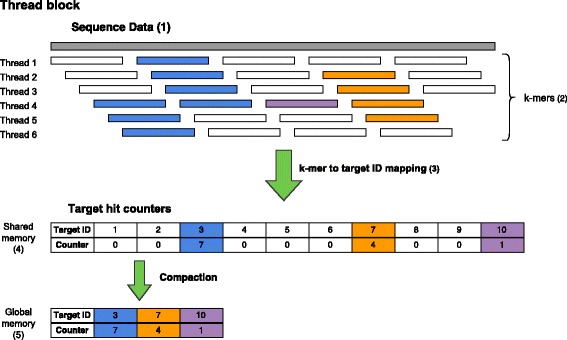
Query kernel. *(1)* The threads load the sequence data, *(2)* construct the k-mers and *(3)* query the database. *(4)* Hits are scored in shared memory first, *(5)* the compact results get transferred to global memory

The query is successful if the *k*-mer has been found in the part of the database currently available to the GPU. In this case a target scores a hit and we increment the hit counter for this target. Here we have to use an atomic add operation, because queries of multiple threads might need to update the counter of the same target. Each atomic operation covers two neighboring counters, because CUDA atomics operate with at least 32 bits and we use 16-bit variables for the counters to minimize shared memory consumption. The query kernel is illustrated in Fig. 4.

After all *k*-mers have been queried, the threads perform a parallel compaction [16] of the target scores in shared memory. This means that all non-zero counters are grouped together to store them efficiently. All counters of value zero offer no additional information and do not need to be saved. The compact results are written to global memory. This strategy greatly reduces the storage space needed as well as the number of accesses to global graphics memory.

In-between the steps we have to synchronize the threads of a block to ensure that all threads have performed the instructions up to this point.

#### Merge kernel

**Fig. 5:**
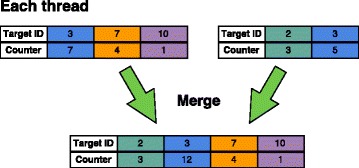
Merge kernel. Each thread combines two compacted target scores for the same sequence into one

The merge kernel is used to combine the compacted target scores. Every call of the kernel merges the results of two GPUs into one, so we can perform a parallel reduction of the results in $\mathcal O(\log _{2} d)$ steps, where *d* is the number of GPUs we use for classification. Each thread in the kernel processes the scores of one sequence. If we have to swap database chunks, the combined scores for each batch are stored in page-locked host memory. After another part has been queried, the results are merged with the previous scores. The merge kernel is illustrated in Fig. 5.

#### Result kernel

**Fig. 6:**
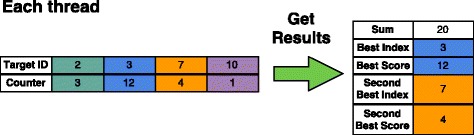
Result kernel. Each thread gets the index and the score of the two best targets for a sequence. It also calculates the sum of all scores for this sequence

The result kernel concludes the GPU stage of the classification process. After all database parts have been examined and the target scores have been merged, we identify the two targets with the highest hit counts for each input sequence. Similar to the merge kernel, we use one thread per sequence to scan the scores (see Fig. 6). After the result kernel has finished, the results are copied back to the host for printing.

### Printing results

After the CPU has scheduled all batches for GPU processing and managed all database swaps, it is ready to print results to an output file. The printing can start after the results of the first batch are available in host memory. Typically the GPUs finish the next batch before the CPU is done printing, so the output process can continue without delay.

In the case that we do not have to swap database parts and each batch is only queried once, we have decided to start printing earlier. After the first batch is scheduled, one CPU thread is immediately assigned to printing while the others continue to process the input file. Thus, we avoid the CPU running idle and further increase the overall execution speed.

While we do not offer all different modes of CLARK, it is still possible to change the sampling factor for loading the database. This can help to increase the speed of the program. Additionally, we provide a light version of our program. It uses a significantly smaller database in order to be executed on systems with a small amount of memory. In our case this involves a CPU with about 4 GB of RAM and a CUDA-enabled GPU with at least 1 GB of graphics memory.

## Results and discussion

### Experimental setup

Experimental results have been obtained by running CLARK (version 1.2.3), cuCLARK and Kraken (version 0.10.5-beta) on a workstation featuring an Intel Xeon E5-2683v4 16-core processor, 128 GB of DDR4 RAM and a CUDA-capable GPU, namely a Pascal-based NVIDIA Titan X with 12 GB of GDRR5X graphics memory. Additionally, we have tested the scalability of cuCLARK with an Intel Xeon E5-2670v2 CPU with 64 GB of DDR3 RAM and four Kepler-based NVIDIA GeForce GTX Titan GPUs each providing 6 GB of GDDR5 video RAM.

To analyze the classification speed of our program we have used simulated metagenome data with different read lengths. The datasets *HiSeq* and *MiSeq* have already been used to evaluate the performance of both Kraken [7] and CLARK [8]. We have furthermore created *wgsim200* and *wgsim250* with wgsim [17] using default settings. While the first two datasets consist of a combination of bacterial whole-genome shotgun reads originating from the corresponding Illumina sequencing platforms, the other two datasets have been created from ten complete bacterial and archaeal genomes randomly picked from the NCBI RefSeq database. The four sets contain 10 million reads each with an average length of 92 base-pairs (bps) for HiSeq, 157 bps for MiSeq and an equal length of 200 bps and 250 bps for all reads in wgsim200 and wgsim250, respectively.

Additionally, we have created a simulated 454 pyrosequencing dataset *art454* with ART [18], using its built-in GS FLX Titanium profile. We have further created a simulated dataset *fqIon* with FASTQSim’s [19] Ion Torrent default characterization. For these two datasets we have used ten randomly selected bacterial and archaeal genomes using a fold coverage of 100x. This resulted in 4,841,892 reads with an average length of 347 bps and 9,626,266 reads with an average 159 bps length, respectively. Note that 454 and Ion Torrent platforms as well as the corresponding simulators produce reads with a much higher variance in length compared to Illumina sequencers. fqIon for example features read lengths ranging from 1 to 264 bps.

### Full mode

For the speed comparison, we have executed read classification with CLARK, cuCLARK, and Kraken for the datasets described above. CLARK is run in full mode and Kraken in default mode with preloaded database. We have used the default *k*-mer length of *k*=31 for all three programs. We have classified against the NCBI RefSeq [20] database of December 2015 consisting of 2785 bacterial genomes. This corresponds to 683 distinct targets at genus-level and 1463 targets at species-level for CLARK and cuCLARK. Runtime results for both taxonomic levels are reported in the Tables 1 and 2.

**Table 1:** Classification speed of CLARK, Kraken, and cuCLARK at species-level measured in terms of 10^6^ classified sequences (reads) per minute

	CPU threads	GPUs	HiSeq Speed	MiSeq Speed	wgsim200 Speed	wgsim250 Speed	art454 Speed	fqIon Speed
CLARK	1	-	1.89	0.88	0.68	0.54	0.29	0.74
CLARK	16	-	15.74	9.74	8.15	6.82	4.05	9.47
Kraken	1	-	2.37	1.33	1.15	0.89	0.64	1.32
Kraken	16	-	12.6	13.74	14.45	11.95	8.75	15.48
cuCLARK	8	1	49.69	42.27	38.02	34.5	26.84	41.01
cuCLARK/CLARK			3.2	4.3	4.7	5.1	6.6	4.3
cuCLARK/Kraken			3.9	3.1	2.6	2.9	3.1	2.7

The bottom two rows report the speedups between cuCLARK over CLARK and Kraken executed with 16 CPU threads

**Table 2:** Classification speed of CLARK, Kraken, and cuCLARK at genus-level measured in terms of 10^6^ classified sequences (reads) per minute

	CPU threads	GPUs	HiSeq Speed	MiSeq Speed	wgsim200 Speed	wgsim250 Speed	art454 Speed	fqIon Speed
CLARK	1	-	1.66	0.75	0.65	0.51	0.26	0.68
CLARK	16	-	13.46	8.96	8.07	6.52	3.9	9.4
Kraken	1	-	2.37	1.33	1.15	0.89	0.64	1.32
Kraken	16	-	12.6	13.74	14.45	11.95	8.75	15.48
cuCLARK	8	1	49.27	41.22	37.23	33.89	25.13	39.93
cuCLARK/CLARK			3.7	4.6	4.6	5.2	6.4	4.2
cuCLARK/Kraken			3.9	3	2.6	2.8	2.9	2.6

The two bottom rows report the speedups of cuCLARK over CLARK and Kraken executed with 16 CPU threads

Classification speed in the tables are measured in terms of 10^6^ classified sequences (reads) per minute. We report the best results of three consecutive runs of the programs with the same input. This was done in order to mitigate runtime differences caused by I/O or cache problems. The measurements do not take the building or loading time of the database into account. The three programs have been executed in single- and multi-threaded configurations. cuCLARK has been executed on a Titan X for single GPU experiments.

For cuCLARK we have decided to process the input files in 16 batches, so the space required for each batch is small enough to leave most graphics memory for the database parts. Since the Titan X’s graphics memory (12 GB) is not sufficient to fit the complete databases of size 38.5 GB (species-level) and 39.91 GB (genus-level) respectively, we need four cycles to process the whole database.

The measurements in Table 1 show that classification speed generally decreases with increasing read length, since longer reads contain more *k*-mers. CLARK and Kraken both scale reasonably well with the number of CPU threads. For instance, CLARK is able to achieve speedups between 8.4-14.0 for the tested datasets when running with 16 threads compared to single-threaded execution. Scaling improves with longer read length.

For cuCLARK we see the same behavior as for CLARK, that classification speeds depend on the sequence length. For our program, however, the differences between the data sets are not as big. For example it is only 18% slower for MiSeq compared to HiSeq.

To further increase the speed of cuCLARK, we use multiple CPU threads to process the input files. We do not change the number of batches, that each file is split into, but instead distribute them among several CPU threads. This way we can provide the GPUs with batches of data at a faster rate which results in a higher overall classification speed. Using eight CPU threads and one GPU, cuCLARK achieves speed-ups of between 3.2 and 6.6 (3.7 and 6.4) when compared to multi-threaded CLARK using 16 CPU threads for species (genus) level classification. The speed-up increases with the read length of the data sets. The genus-level classification speeds for CLARK and cuCLARK are slightly slower than for species level. This is caused by the larger database, which leads to longer queries on average.

If executed with a low number of threads, Kraken is 1.3-1.8 times faster than CLARK at the species- or genus-level with the same thread count. For MiSeq, wgsim200„ wgsim250 and fqIon Kraken using 16 threads is still 1.4-1.8 times faster than CLARK with the same number of threads. Kraken performs especially well for art454, where it is 2.2-2.5 times faster than CLARK in single- and multi-threaded execution. cuCLARK is 2.6-3.9 (2.6-3.1) times faster than Kraken running with 16 CPU threads for species (genus)-level classification.

### Dependency On database size

When loading the database with a sampling factor of *s*=6, we can investigate the performance on smaller database sizes. A sampling factor of *s*=1 is equivalent to the default behaviour of loading the whole database. Using a sampling factor *s*>1 whole buckets are skipped and thus many queries result in an immediate miss. As a result, the average query speed increases. Due to this, CLARK is able to improve its runtime by 58% for the execution with 16 threads. Moreover, it needs about 52 GB less RAM for the classification.

For cuCLARK the main benefit of the smaller database is that we can completely fit it into graphics memory. Thus, we only need to query the batches once and do not need to swap database parts on-the-fly. We observe that the gain in speed for the single-threaded execution is relatively small. Therefore, the speed of cuCLARK is obviously bound by I/O operations executed on the CPU. For this reason we have implemented the early printing feature for the multi-threaded mode. During concurrent execution we can hide parts of the time needed for the file output and thus increase overall speed. Using multiple CPU threads we obtain a speedup of 51%-53% instead of only 37% improvement over the single-threaded version. Note that printing early is only possible if we do not need to query the batches against another database part.

Among all sampling factors the main memory consumption of CLARK is in general reasonably higher than cuCLARK’s although they both store the same database entries and consequently produce compliant results. This can be explained as follows. CLARK’s nested data structures require more bytes for the storage of individual buckets due to sub-optimal memory alignment. In fact we use less than half the amount of RAM as CLARK by consecutively storing the buckets in one contiguous array. This reduction in memory consumption can be observed for the whole database as well as the sampled one (see Table 3).

**Table 3:** Classification speed for different sampling factors at species-level for the HiSeq dataset in terms of 10^6^ sequences/min and corresponding main memory consumption

HiSeq	CPU threads	GPUs	Speed	RAM usage	GRAM usage
CLARK (s=1)	16	-	15.74	86.5 GB	-
CLARK (s=6)	16	-	24.8	34.7 GB	-
Kraken	16	-	12.6	73.8 GB	-
cuCLARK (s=1)	1	1	44.26	39.9 GB	9.7 GB
cuCLARK (s=1)	8	1	49.69	39.9 GB	9.7 GB
cuCLARK (s=6)	1	1	60.51	14.1 GB	11.9 GB
cuCLARK (s=6)	8	1	75.96	14.1 GB	11.9 GB

### Light versions

Furthermore, we have classified the data sets with the light versions of CLARK and cuCLARK, which construct a much smaller database and need less than 4 GB of RAM. We have used the full mode for CLARK light and default *k*-mer length (*k*=27). For cuCLARK light the reduced database is smaller than 720 MB and leaves enough space for batch data even on graphics cards with only 1 GB of memory. The resulting speeds for the light versions are presented in Table 4.

**Table 4:** Classification speed comparison between CLARK light and cuCLARK light at species-level in terms of 10^6^ classified sequences/min

	CPU threads	GPUs	HiSeq Speed	MiSeq Speed	wgsim200 Speed	wgsim250 Speed	art454 Speed	fqIon Speed
CLARK light	1	-	5.85	3.06	2.64	2.08	1.08	2.66
CLARK light	16	-	27.32	19.48	16.47	13.95	8.21	18.45
cuCLARK light	8	1	75.46	69.76	62.62	59.23	54.12	65.27
cuCLARK light/CLARK light			2.8	3.6	3.8	4.2	6.6	3.5

The bottom row reports the speedup of cuCALRK light compared to CLARK light executed with 16 CPU threads

### Scalability

In order to investigate the scalability of our implementation we have run cuCLARK on multiple GPUs attached to one workstation. The up to four Kepler-based GeForce GTX Titans each provide 6 GB of video RAM. The increased amount of total memory allows for the querying of a larger part of the database at once resulting in less cycles to process the whole database. Using a single GPU we need seven cycles to process the whole species database, four cycles for two GPUs and two cycles for four GPUs, respectively.

**Fig. 7:**
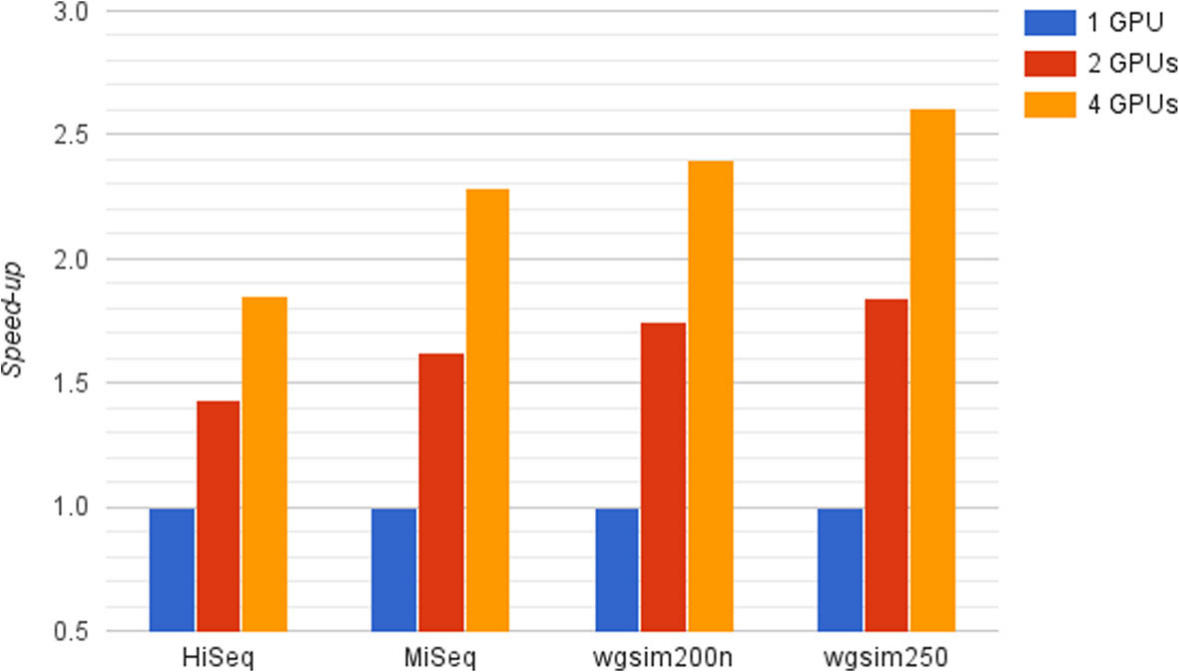
cuCLARK GPU scaling. Speed-up of cuCLARK from one to two and four GPUs for the different datasets. Four NVIDIA GeForce GTX Titan with 6 GB graphic memory were used for this experiment

Since I/O is independent of the number of used GPUs, the higher speeds observed when using multiple GPUs result from the simultaneous processing of database parts. This leads to a reduced number of work steps per graphics device. The speed-up increases with the read length of the datasets, as depicted in Fig. 7. Due to the increased length of reads the classification can attain a higher efficiency. We achieve a speedup of 1.4 to 1.8 with two GPUs and a speedup of 1.8 to 2.6 with four GPUs compared to the classification on one GPU.

## Conclusion

The rapidly growing field of metagenomic sequencing creates an urgent need for exceedingly fast and precise computational tools to analyze the ever-increasing amount of recorded data. In this context, taxonomic read assignment is a frequently repeated and important task. Thus, novel short read classifiers relying on efficient algorithmic design specifically suited for the execution on modern hardware accelerators are of high importance for academic research and commercial bioinformatics solutions.

We have introduced cuCLARK, a hybrid metagenomic read classifier executed on multi-core CPU and massively parallel GPUs. It can assign taxonomic labels at the speed of up to 50 million reads per minute using a single GPU, while producing identical results to the established CLARK tool.

The execution speed can be maximized if the available graphics devices provide enough memory to fit the entire database of discriminative *k*-mers. In this case asynchronous read classification on GPUs can be accelerated to an extent that the program’s speed is solely limited by the I/O operations executed on the CPU. Therefore, multi-threaded processing of input and output allows for the further increasing of performance since CPU-based I/O can be efficiently overlapped with GPU-based classification.

If cuCLARK is executed on GPUs with relatively small video RAM sizes, it needs several classification cycles to process the whole database. This leads to lower speeds, because the input sequences need to be queried successively against multiple parts of the database. In our experiments we needed four cycles, when using a GPU with 12 GB of memory and the NCBI RefSeq database of December 2015. In this configuration achieved speedups for species-level classification range between 3.2 and 6.6 (2.6 and 3.3) on a single Titan X GPU compared to CLARK (Kraken) when executed with 16 threads on a Xeon E5-2683v4 16-core CPU.

The light mode of cuCLARK is able to reach even higher speeds than the full version. It proved to be a superior alternative to CLARK light if the user can provide a suitable graphics device. cuCLARK light is up to 6.6 times faster on a single GPU than CLARK light using 16 CPU threads.

As our experiments have shown, the classification speed of our program heavily correlates with the amount of available video RAM and the memory bandwidth. Future accelerator cards from the professional Tesla branch, e.g the to be released Tesla P100 chip, will feature 16 GB of high bandwidth stacked memory [21]. This novel memory type allows for significantly higher bandwidths in comparison to current GDDR5 and GDDR5X modules. Thus a possible direction of future research could be the investigation of the impact of high bandwidth memory on the performance of hash-based short read classifiers.

## Abbreviations

bps: Base pairs
CPU: Central processing unit
CUDA: Compute unified device architecture
GPU: Graphics processing unit
LCA: Lowest common ancestor
NCBI: National center for biotechnology information
NGS: Next-generation sequencing
PCIe: Peripheral component interconnect express
